# Estimating Aggregate Environmental Risk Score in Psychiatry: The Exposome Score for Schizophrenia

**DOI:** 10.3389/fpsyt.2021.671334

**Published:** 2021-05-28

**Authors:** Lotta-Katrin Pries, Gamze Erzin, Bart P. F. Rutten, Jim van Os, Sinan Guloksuz

**Affiliations:** ^1^Department of Psychiatry and Neuropsychology, School for Mental Health and Neuroscience, Maastricht University Medical Center, Maastricht, Netherlands; ^2^Department of Psychiatry, University of Health Sciences Ankara Diskapi Training and Research Hospital, Ankara, Turkey; ^3^Brain Centre Rudolf Magnus, University Medical Center Utrecht, Utrecht, Netherlands; ^4^King's Health Partners, Department of Psychosis Studies, King's College London, Institute of Psychiatry, London, United Kingdom; ^5^Department of Psychiatry, Yale University School of Medicine, New Haven, CT, United States

**Keywords:** exposome, prediction, risk, environment, schizophrenia, psychosis, gene-environment

## Abstract

To understand the role of environment in the pathoetiology of psychosis spectrum disorders, research has thus far mainly investigated the effects of single exposures in isolation, such as the association between cannabis use and schizophrenia. However, this approach fails to acknowledge the complexity of the exposome, which represents the totality of the environment involving many exposures over an individual's lifetime. Therefore, contemporary research adopting the exposome paradigm has aimed at capturing the combined effect of different environmental exposures by utilizing an aggregate environmental vulnerability score for schizophrenia: the exposome score for schizophrenia. Here, we attempt to provide a comprehensive overview of studies applying the exposome score for schizophrenia. First, we describe several approaches estimating exposomic vulnerability for schizophrenia, which falls into three categories: simple environmental sum scores (sum of dichotomized exposures), meta-analysis-based environmental risk score (sum scores weighted by estimates from meta-analyses), and the exposome score (sum score weighted by estimates from an analysis in an independent training dataset). Studies show that the exposome score for schizophrenia that assumes interdependency of exposures performs better than scores that assume independence of exposures, such as the environmental sum score and the meta-analysis-based environmental risk score. Second, we discuss findings on the pluripotency of the exposome score for schizophrenia and summarize findings from gene-environment studies using the exposome score for schizophrenia. Finally, we discuss possible scientific, clinical, and population-based applications of exposome score for schizophrenia, as well as limitations and future directions for exposome research to understand the etiology of psychosis spectrum disorders.

“*Those who study the complex interplay of cause and effect in the history of the Universe say that this sort of thing is going on all the time, but that we are powerless to prevent it. ‘It's just life,’ they say (**1**)*.”

## Introduction

Psychosis spectrum disorders (PSD) have a complex pathoetiology involving genetic and environmental factors. To understand the genetic background of PSD, research moved from hypothesis-driven candidate gene studies to agnostic genome-wide association studies (2). Eventually, increasing availability of low-cost genome-wide genotyping and larger samples have made it possible to calculate a weighted sum score of trait alleles that captures molecular measures of genetic vulnerability for schizophrenia: polygenic risk score for schizophrenia (PRS-SCZ) (2). However, studies indicate that the molecular genetic vulnerability for schizophrenia captured by PRS-SCZ only explains 7.7% of the variance in liability attributable to schizophrenia, with the SNP-based heritability being around 24% (2). These are considerably below the 60–80% heritability estimates previously demonstrated in family and twin studies (3–6). This “heritability gap” is a strong indicator that at least part of the pathoetiology for schizophrenia may be explained by environmental factors (7), besides other explanation such as gene-gene interactions.

Traditionally, epidemiological studies investigating the contribution of the environment to PSD have focused on distinct environmental factors, each investigated in isolation (8). Umbrella reviews show that environmental factors such as childhood adversity (e.g., sexual or physical abuse), cannabis use, urbanicity, social defeat (i.e., migration status, ethnical minority), obstetric complications, and season of birth are associated with PSD (9, 10). Furthermore, research shows that the exposure to a higher number of environmental factors is indicative of the outcome severity (11, 12).

However, these approaches are not designed to capture the exposome, which is the entirety of environmental factors an individual is exposed to throughout their life (8, 13). Furthermore, the dependency (i.e., correlation) between different environmental exposures should be taken into account. Research investigating the moderating and mediating effects of environmental exposures has shown that the network of environmental exposures works in concert to give rise to psychosis expression (14–16). Environmental factors are bidirectionally interlinked, such that cannabis use is associated with childhood adversity (12), which is among other exposures associated with stressful events later in life (17). The effects of urbanicity variables (e.g., population density, social fragmentation and deprivation) may be confounded or modified by individual level factors such as cannabis use, social adversity, exclusion, and discrimination that are observed more frequently in large cities than rural areas (18). Different childhood adversity types, such as sexual and physical abuse, are inter-correlated, with co-occurrence being indicative of the severity of the outcome (19, 20). Around 47.9% of individuals who are exposed to sexual abuse will be exposed to re-victimization later in life (21). A study investigating the “vibration of effects” (i.e., the amount of fluctuation of results from different model specifications) showed that results in analytical models of exposures were dependent on the model specifications, such as the inclusion of different sets of variables (8). These findings show that environmental vulnerability for PSD cannot be understood in isolation. Therefore, the exposome framework has recently been adopted in environmental research.

In this mini review, we aim to discuss findings from research investigating the exposome in relation to PSD, with a particular focus on estimating an aggregate environmental vulnerability score for schizophrenia (exposome score for schizophrenia: ES-SCZ) (22). We will first introduce the different approaches for estimating exposomic liability for schizophrenia and discuss their advantages and limitations. We will then discuss the pluripotency of the exposome score for schizophrenia and findings from gene-environment studies using the ES-SCZ. We will discuss the potential utility of the ES-SCZ at research, clinical, and public health settings. Finally, we will outline future directions for exposome research to dissect the complexity of environment in the pathogenesis/etiology of PSD.

## Estimating an Aggregate Environmental Risk Score for Schizophrenia

To capture the combined effect of different environmental exposures, researchers used simple summation scores that are generated by adding up each dichotomized environmental variable (11, 12, 23, 24). These studies revealed that such a cumulative environmental risk was associated with increased severity of psychopathology and clinical features. However, the simple summation of exposures as a risk score fails to capture the varying degrees of risk attributable to each exposure for psychosis liability. For instance, peer bullying, emotional abuse, and hearing impairment are all associated with an increased likelihood for schizophrenia diagnosis. However, the meta-analytical estimates suggest that the odds ratio for bullying (OR = 2.39) (25) is smaller than those for other environmental exposures, such as emotional abuse (OR = 3.40) (25) and hearing impairment (OR = 3.15) (26). In this regard, by handling exposures equally, the simple summation of exposures fails to acknowledge the magnitude of each exposure's risk.

To take into account different weights of environmental exposures, previous work used coefficients derived from meta-analyses to calculate weighted sum scores, albeit with different sets of variables (22, 27, 28). Padmanabhan et al. (28) were the first to use meta-analytical estimates to calculate a weighted sum score of dichotomized environmental exposures including childhood adversities (i.e., sexual and physical abuse, neglect, and parent death), cannabis abuse, advanced paternal age, urban upbringing, obstetric and perinatal complications, and winter-birth associated with schizophrenia. The “polyenvironmental risk score” explained 14% of the variance for psychosis conversion in young relatives of individuals diagnosed with schizophrenia (28). A similar estimate of *R*^2^= 13% for a meta-analysis-based environmental risk score (including childhood adversities, hearing impairment, bullying, cannabis use, and winter-birth) was found for the diagnosis of schizophrenia spectrum disorder in a case-control study (22), whereas another study indicated a lower *R*^2^ of around 4.6% (27) using a slightly different approach with estimates derived from meta-analyses of ordinal and dichotomized variables in a simulated dataset. Further, using the same approach as the latter researchers, in a sample with first episode psychosis patients and healthy controls, this meta-analyses-based environmental risk score explained 8.4% of the variance in case-control status (29). Although the meta-analytical approach takes into account different weights of environmental exposures, it fails to embrace the interdependency (i.e., correlation) between exposures, similar to the environmental sum-score.

To overcome these limitations, Pries et al. (22) constructed the exposome score for schizophrenia (ES-SCZ) that took correlations between exposures into account. In this study, several prediction models [logistic regression (LR), Gaussian Naive Bayes (GNB), the least absolute shrinkage and selection operator (LASSO), and Ridge penalized classification (RIDGE)] were applied in a case-control sample to derive weighted risk per exposure including five domains of childhood adversities (emotional, sexual and physical abuse along with emotional, and physical neglect), bullying, cannabis use, winter-birth, and hearing impairment. In an independent validation dataset, the estimates from each model were subsequently used to calculate the weighted ES-SCZ along with the simple sum score and an environmental risk score using estimates from meta-analyses. The environmental risk scores derived from the models that took interdependencies (LR, LASSO, RIDGE) between environmental exposures into account performed better in regard to accuracy and sensitivity, in comparison to those assuming independent effects of each exposure: the score based on GNB estimates, the simple summation, and meta-analytical estimates. Given equally good model performances of LR, LASSO, and RIDGE, the ES-SCZ was calculated based on estimates from the more accessible model (LR) and were used for subsequent analyses. For a comparison of the sum score, the meta-analysis-based environmental risk score, and the ES-SCZ in regard to limitations and performance see Table 1. The ES-SCZ was able to discriminate patients with a diagnosis of schizophrenia, their siblings, and healthy controls in the validation dataset. The follow-up study using data from an independent general population cohort likewise revealed that the performance of ES-SCZ for identifying clinical psychosis diagnosis was better than the environmental sum score and the environmental score derived from meta-analytical estimates (30). Furthermore, within a large international sample with patients diagnosed with schizophrenia and healthy controls, the ES-SCZ explained 28% of the variance (Nagelkerke's pseudo *R*^2^) in case-control status and 33% after adjusting for age, sex, and country, whereas the PRS-SCZ explained 15% (adjusted for 10 principal components) and 20% after additionally adjusting for age, sex, and country (31).

**Table 1:** overview and comparison of environmental aggregate scores.

**Environmental aggregate scores**	**Limitations**	**ROC**	**Accuracy**	**Explained variance**
Sum score	1. Does not take into account different weights of environmental exposures 2. Does not take into account correlation between environmental exposures 3. Limited to assessed environmental exposures in the test sample	0.71	0.67	17%
Meta-analysis-based score	1. Does not take into account correlation between environmental exposures 2. Limited to assessed environmental exposures in samples used in the meta-analyses and the test sample	0.69	0.62	13%
Exposome score for schizophrenia	1. Limited to assessed environmental exposures in the training sample and the test sample	0.73	0.68	21%
				

*Results are from Pries et al. (22) analyses associating a simple sum score, a score based on meta-analyses, and ES-SCZ including the same environmental exposures (i.e., childhood adversities, cannabis use, hearing impairment, and winter-birth) with (schizophrenia-)case-control status; ES-SCZ, exposome score for schizophrenia; ROC, area under the receiver operating characteristic curve, Explained variance: Nagelkerke's pseudo R^2^*.

## Is the Exposome Score for Schizophrenia Phenotype-Specific?

It has been argued that environment (e.g., childhood adversity) impacts psychosis expression across the psychosis spectrum, from subclinical psychotic experiences to most severe clinical outcomes, such as schizophrenia (25). In accordance, findings revealed that ES-SCZ was associated not only with schizophrenia diagnosis but also with schizotypy in healthy comparisons and unaffected siblings (31). Furthermore, in the general population, ES-SCZ was associated with the psychosis risk strata: the higher the ES-SCZ, the greater the psychosis risk level (32).

Evidence indicates that exposomic vulnerability for schizophrenia is not only etiologically continuous with psychosis spectrum but also associated with pluripotent psychopathology that cuts across traditional diagnoses (e.g., depression and anxiety). Previous research showed that environmental factors that have previously been associated with psychosis and that are incorporated in the ES-SCZ, such as childhood adversities and cannabis use, are associated with expression of mental and physical health problems, as well as with multidimensional expression of psychopathology (8, 13, 19). In line with these findings, a recent study found that ES-SCZ was temporally linked to general mental and physical health outcomes in the general population (33). Similarly, another study revealed that ES-SCZ was also associated with increased risk for various mental disorders (e.g., depression, anxiety, and alcohol use disorders), personality traits (i.e., neuroticism and extraversion), and medical complaints including migraine, asthma, and ulcers (30).

## Utilizing Exposome Score for Schizophrenia to Explore Diathesis–Stress Model

Epidemiological studies consistently show that environmental factors have an influence on mental health outcomes, with the degrees of impact varying across people. These differences may be explained by the diathesis-stress theory (34) that posits that a combination of genetic and environmental factors modulate the development of more severe psychopathology. More specifically, genetic and early environmental vulnerabilities may make an individual more susceptible to environmental exposures later in life.

In accordance with the diathesis-stress model, a recent study showed that childhood adversity and cannabis use interacted with PRS-SCZ increasing the likelihood to develop schizophrenia (35). Guided by these findings, the follow-up study analyzed the interaction between PRS-SCZ and ES-SCZ in association with schizophrenia diagnosis using a case-control design and in association with schizotypy in siblings of patients with schizophrenia and healthy controls (31). Findings from these studies, showing that the relative excess risk due to the interaction were above 2, suggest that “mechanistic” interaction drives the liability for schizophrenia. This means that both genetic and environment risk should be present for some individuals to develop schizophrenia (36). In line with these findings, PRS-SCZ showed a “mechanistic” interaction with a meta-analyses-based environmental risk score in a sample of patients with first episode psychosis and healthy controls (29). Overall, findings from these studies support the idea that genomic and exposomic vulnerability interactively influence psychosis expression across the spectrum from the “soft-phenotypes” detected in the general population to clinical disorders.

In accordance with the two-hit model as discussed above, early environmental pre-disposition, such as childhood adversity, may moderate the response to environmental exposure later in life, such as stressful life events. A recent population-based prospective cohort study examining the two-hit model revealed that environmental pre-disposition for schizophrenia captured by the ES-SCZ increased the detrimental impact of recent stressful life events on mental and physical health outcomes, thereby suggesting an environment-environment interaction (33). These findings highlight the need to investigate the interplay between early environmental exposure load and temporally proximal environmental exposures to better understand the complex etiology of PSD.

## Future Directions

Embracing the exposome paradigm opens up the opportunity for scientific, clinical, and population-based endeavors. Among others, a cumulative environmental metric, such as the ES-SCZ, may be used for environmental error adjustment in statistical modeling in epidemiological studies. This may improve the statistical power for epidemiological studies, which in turn, may help to further dissect the etiology of psychopathology. Furthermore, combining exposomic and genomic vulnerability for schizophrenia might deepen our current understanding of the complex etiology of PSD. In this regard, to test gene-environment interaction, additive models might be preferable to multiplicative models as they optimally capture biological synergy (37) and may help to form public health decisions in accordance with the sufficient cause framework (38, 39).

Eventually, ES-SCZ may be used for selective risk-enrichment to target selective smaller samples with heightened environmental risk, ultimately giving the opportunity to conduct expensive, experimental or time-consuming trajectory studies that are aimed to explore risk and resilience mechanisms. Furthermore, future approaches incorporating exposomic information within electronic health records or health screenings may potentially help healthcare providers identify vulnerable individuals who need further support during stressful periods. In this regard, as the effect of ES-SCZ is non-specific, it is plausible to argue that in these risk-enriched samples, it might be more fruitful to evaluate the trajectory of multidimensional psychopathology rather than the trajectory of traditional diagnostic categories such as schizophrenia.

Although the ES-SCZ offers great opportunities for future studies, several limitations and possible improvements need to be mentioned. Successful application of aggregate environmental scores such as the ES-SCZ are dependent on the availability of similar assessment of environmental exposures in the training and validation datasets. In this regard, ES-SCZ was limited to nine environmental exposures that were available in the training and validation datasets; and therefore, the application of ES-SCZ has been confined to cohorts with similar assessment protocols for exposures. Of note, these limitations are not unique to ES-SCZ but also registered in environmental scores that use estimates derived from other studies, such as meta-analyses. In addition to the nine environmental exposures included in the ES-SCZ, an extended coverage of exposome domains that include correlates such as psycho-social, lifestyle [e.g., smoking (40)], chemical, peripheral (e.g., inflammatory) markers (41), malnutrition (especially prenatal), vitamin D levels (40), and metabolic changes (42) would provide a more complete ES-SCZ and might benefit the predictive power of the ES-SCZ. However, it should be noted that this would decrease the applicability of the ES-SCZ as most of these markers and environmental correlates are not available uniformly across the cohorts; and some of them, such as pre-, perinatal period adversities, are challenging to reliably collect retrospectively without access to detailed medical records that have been recorded at birth. Future cohort studies should pay specific attention to collecting consistent data in deeply phenotype cohorts to increase data harmonization efforts (13).

Adding pairwise interactions of exposures may further improve the performance of ES-SCZ. However, with the inclusion of different correlates of PSD and pairwise interactions, more complex modeling (e.g., penalized classification models) of these exposures might be necessary (22). Furthermore, so far, the ES-SCZ represents vulnerability for schizophrenia. Other factors that “protect” or buffer vulnerability may also be identified and investigated (43–45). Future studies will need to evaluate how vulnerability, protective, and resilience factors work together forming the exposome.

As known exposures impacting PSD are still limited and no major advances in the field have been observed recently, agnostic exposure-wide analytical approaches that take into account of inter-correlation may provide further understanding of other, so far, unknown factors that also cover other domains of the exposome such as internal (e.g., inflammation) and external (e.g., chemical, lifestyle, psycho-social) correlates (13). In this regard, similar to previous studies in other phenotypes such as HIV (46), diabetes (47), depression (48), and childhood behavior (49), our research group is currently conducting a systematic exposome-wide investigation of correlates of psychosis expression in the UK Biobank.

Research on exposomic vulnerability has been largely conducted in cross-sectional designs. Prospective analyses may be especially fruitful to disentangle the effects of the exposome on the trajectory of mental health outcomes over time. Studies found that the proximity of stressors can be an important factor for determining their impact on psychopathology (33). Furthermore, stressors during sensitive periods of neurodevelopment may play a crucial role in determining the trajectory of mental health outcomes (40). Therefore, approaches that take into account of time-sensitive effects are warranted to advance this new field of exposomic research.

## Conclusion

Identifying modifiable environmental factors is crucial for improving mental health outcomes. The exposome paradigm may further advance the progress to gaining insight into the complex dynamic network of environment underlying the pathoetiology of psychosis spectrum disorder. Furthermore, integrating individual-level environmental vulnerability (i.e., ES-SCZ) into risk models may offer potential benefits. Future research should aim at expanding and refining exposomic liability for psychosis by identifying other, so far, unknown exposures, integrating resilience factors, and employing more complex time-sensitive modeling of exposures.

## Author Contributions

L-KP and SG conceived the idea of this review and developed the outline. L-KP wrote the first draft of this manuscript. SG provided critical revision of the manuscript for important intellectual content. BR, GE, and JO reviewed the manuscript and provided comments/suggestions. All authors contributed to the final version and have approved the final manuscript.

## Conflict of Interest

The authors declare that the research was conducted in the absence of any commercial or financial relationships that could be construed as a potential conflict of interest.
